# Optical Fiber LSPR Biosensor Prepared by Gold Nanoparticle Assembly on Polyelectrolyte Multilayer

**DOI:** 10.3390/s100403585

**Published:** 2010-04-08

**Authors:** Yunliang Shao, Shuping Xu, Xianliang Zheng, Ye Wang, Weiqing Xu

**Affiliations:** 1 State Key Laboratory of Supramolecular Structure and Materials, Jilin University, Changchun 130012, China; E-Mails: yunliangshao@gmail.com (Y.S.); xusp@jlu.edu.cn (S.X.); zhengxl@jlu.edu.cn (X.Z.); xiaoye2067@gmail.com (Y.W.); 2 Department of Materials Science, Jilin University, Changchun 130012, China

**Keywords:** localized surface plasmon resonance (LSPR), metal nanoparticle, optical fiber sensor, self-assembly, immuno detection

## Abstract

This article provides a novel method of constructing an optical fiber localized surface plasmon resonance (LSPR) biosensor. A gold nanoparticle (NP) assembled film as the sensing layer was built on the polyelectrolyte (PE) multilayer modified sidewall of an unclad optical fiber. By using a trilayer PE structure, we obtained a monodisperse gold NP assembled film. The preparation procedure for this LSPR sensor is simple and time saving. The optical fiber LSPR sensor has higher sensitivity and outstanding reproducibility. The higher anti-interference ability for response to an antibody makes it a promising method in application as a portable immuno-sensor.

## 1. Introduction

Research on the preparation of localized surface plasmon resonance (LSPR) optical fiber sensors utilizing the association of noble metal nanoparticle (NP) LSPR properties and optical fibers has become a popular subject. The localized electromagnetic field around metal surfaces is very sensitive to environmental refractive indexes [1,2]. Environmental changes at the interfaces between media and metals can be traced by the changes of metal LSPR characteristics. The coupling of LSPR sensors and optical fibers bring several advantages. First, multiplying the total reflection of light transmission in the optical fiber excites a sample many times, so the detection sensitivity of analytes is expected to be improved. Secondly, the penetration depth of evanescent field on the sidewall of unclad optical fiber is only 100–200 nm [3]. Therefore, the sensor is only sensitive to the change of the environment nearby the optical fiber surface. In this way, the background noise can be greatly suppressed. Thirdly, the optical fiber sensor prepared based on the metal NP LSPR effect has the benefits of real-time detection, fast response and easy modification[4].

At present, the preparation of metal NP sensing films for optical fiber sensors mainly makes use of silane as a linking agent to assemble metal NPs [5–11]. It was required to make optical fibers hydroxylated first. Then the optical fibers were immersed in the (3-mercaptopropyl) trimethoxysilane (MPTMS) toluene solution for 8 h and a gold colloid for 5 h [5]. Alternatively, the optical fibers were immersed in the (3-aminopropyl) trimethoxysilane (APTMS) ethanol solution with little acetic acid for 10 min, and then left at 120 °C to complete silylation, and finally assembled metal NPs [7]. The complex operation and lengthy manipulation limit the application of optical fiber LSPR sensors.

In this paper, we utilize the method of polyelectrolyte (PE) alternate assembly of metal NPs to prepare the optical fiber LSPR sensor [12]. Our goal was to simplify the operating procedures for the preparation of optical fiber LSPR sensors and to quickly prepare a high-capacity sensing layer. The approach of the PE alternate assembly of metal NPs can achieve this goal. We monitored both the etching process of optical fibers and the assembly process of metal NPs. The sensitivity of the optical fiber LSPR sensor prepared by this method was evaluated and compared with other sensors. In addition, the optical fiber LSPR sensor was utilized for the immunoassay of goat anti-rabbit IgG.

## 2. Results and Discussion

**Figure 1.**
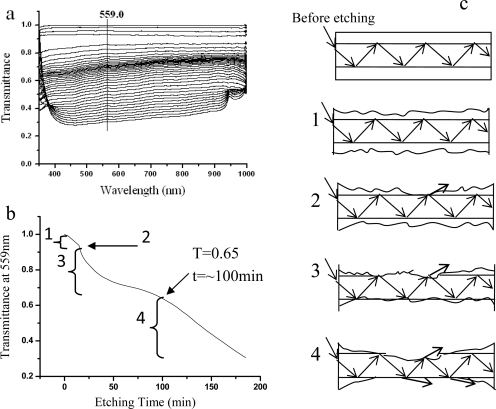
(a) The transmission spectra at different etching time. (b) The transmittance at 559.0 nm as a function of the HF etching time. (c) The schematic drawing of the fiber optic at different etching stages.

**Scheme 1.**
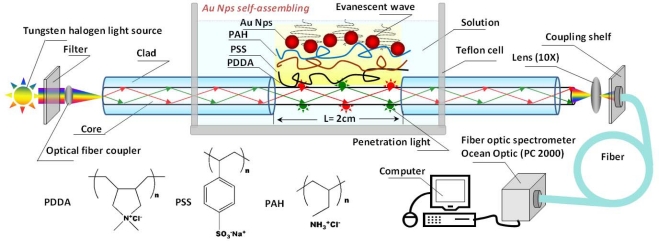
The experimental setup of the optical fiber LSPR sensor.

Scheme 1 shows the setup of the optical fiber LSPR sensor. The optical fiber we used is quartz clad silica fiber (P600-4 UV-VIS, Ocean Optics Co.). The incident light was focused on the fiber core and limited in the core. A 2-cm long the cladding layer of the optical fiber was removed through the etching treatment by HF solution. Figure 1a presents the serial transmission (I/I_0_) curves collected at different etching time. The transmission at 559.0 nm was plotted with the etching time (Figure 1b). The transmittance dropped gradually. The etching process can be divided into four stages (1–4 in Figure 1b,c). Stage 1 presents the start of light leakage during the initial etching process. Stage 2 shows the moment where the transmittance had a sudden drop, which indicates that the etching reached the fiber core. The etching around the core continued in Stage 3 and the penetrated light largely increased. The cladding layer was completely removed in Stage 4, and more and more light leaked. We usually stopped the etching process prior to Stage 4. At this moment, the cladding layer was completely removed and most of the core was kept, thus ensuring the optical fiber has the greatest light capacity and mechanical strength. The corresponding transmittance is 0.65.

For immobilizing a LSPR sensing layer, a trilayer PE structure was chosen as a linker for self-assembly of Au NPs. The gold NPs were immobilized by the electrostatic interaction. Since the fiber was functionalized by negative charged hydroxy groups, the first PE layer we used was PDDA, a strong cationic PE. After the PDDA assembly, the fiber surface was positive charged. Then the ionic PE (PSS) and the comparatively weak cationic PE (PAH) were sequentially assembled. The citrate stabilized Au NPs were finally immobilized onto the outmost PAH layer.

The whole processing takes less than four hours. It includes 100 min for the etching of the optical fiber (the etched optical fiber can be repeatedly used), 40 min for the hydroxylation of optical fiber by H_2_SO_4_/H_2_O_2_ solution, and 75 min for the assembly of PDDA/PSS/PAH/Au (15 min for PDDA, 10 min for PSS, 10 min for PAH and 40 min for Au NPs). This method is very time-saving compared with the assembly of Au NP *via* the silane coupling agent (about 13 h) [5].

**Figure 2.**
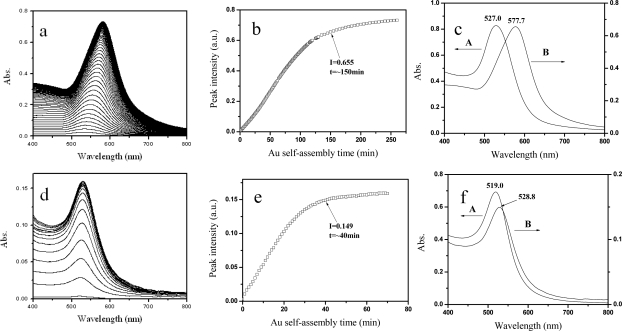
The absorption spectra of Au NP assembled films at different assembly time. (b) and (e); the LSPR peak intensity as a function of different assembly times. The assembly time is about 150 min for 48-nm Au and 39 min for 23-nm Au. (c) and (f); the absorption spectra before (A) and after the Au NP assembly (B). (a)-(c); 48-nm Au. (d)-(f); 23-nm Au.

It was reported that the responses of sensitivities of LSPR sensors varied when different sized metal NPs were employed [13]. In present study, we explored two sizes of Au NPs (*d* = 48 and 23 nm). A series of LSPR spectra of Au NPs in their assembly process were recorded (Figure 2a,d). A red shift of its LSPR band and an increase of peak intensities were observed. This means that the quantity of Au NPs immobilized on the optical fiber sidewall increased and the interparticle spacing between Au NPs reduced [14]. Figure 2b and e show the trends of the LSPR peak intensities as a function of the Au NPs assembly time. At the beginning, the adsorbing process was fast, because there were a great number of active spots on the PE trilayer for the adsorption of Au NPs. Then the peak intensities increasd slowly. There might be two reasons to explain this phenomenon: one is the reducing of active spots on the trilayer PE matrix, and the other is that the interparticle electrostatic repulsion arose from more and more Au NPs. For the 48-nm Au colloid, when the assembly time is about 150 min, its peak intensity is 0.655 and the peak position is 577.7 nm. For the 23-nm Au colloid, the peak intensity is 0.149 and the peak position is 528.8 nm when the assembly time is about 39 min. It indicates that the size of Au NPs could influence the assembly time. The peak positions of the Au NP assembled films had 50.7 nm (for the 48-nm Au NPs) and 9.8 nm (for the 23-nm Au NPs) red shifts relative to their original Au colloids, respectively (Figure 2 c,f).

**Figure 3.**
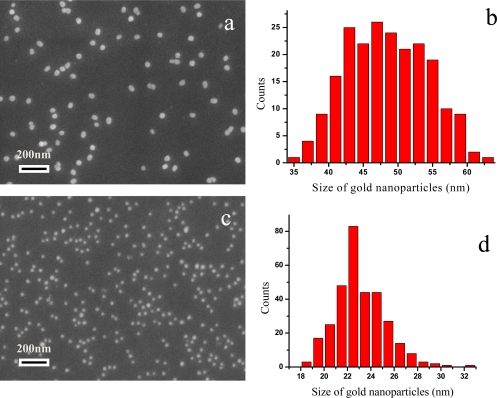
(a) and (c) The SEM images of the Au NP assembled films prepared with two different sizes of Au NPs; (b) and (d) The size distribution of Au NPs in (a) and (c).The diameter of (b) and (d) is 48 ± 6 nm and 23 ± 2 nm, respectively.

SEM was used to characterize the Au NP films deposited on the trilayer PE substrates. Figure 3a and c show the SEM images of the Au NP films prepared by different sized Au NPs. The assembled Au NPs are spherical in morphology, with the size of 48 ± 6 nm and 23 ± 2 nm. The distribution of the Au NPs on the assembled film is uniform. No aggregates of Au NPs can be seen. The coverage fraction of colloid A and B in the film is 6.934% ± 1.625 and 5.686% ± 0.946, for 48-nm and 23-nm Au, respectively.

It is well known that the aggregation of Au NPs will broaden and decrease the LSPR band (at ∼520 nm) and generate multipolar resonance bands (within 600–700 nm) in the long wave region [15]. The aggregation will strongly affect the LSPR sensing performance and experimental reproducibility. Both the LSPR spectra of Au NP sensing films and SEM results indicate that the Au NPs prepared by the present method were well dispersed. Obviously, the multilayered PEs play an important role in the Au NPs assembly. The three consecutively alternate PE layers are composed of a strong cationic PE as the first layer, an ionic PE as the second layer and a comparatively weak cationic PE as the third layer [12]. This trilayer structure would limit the PE layer interpenetration to a certain degree [16], which would lead to the relative uniform surface charge distribution on the PE multilayer for Au NP assembly. In present study, both well-dispersed Au NPs on SEM images and single plasmon band in LSPR spectra agree on that the trilayer PE structure is favorable for the preparation of LSPR sensing films.

**Figure 4.**
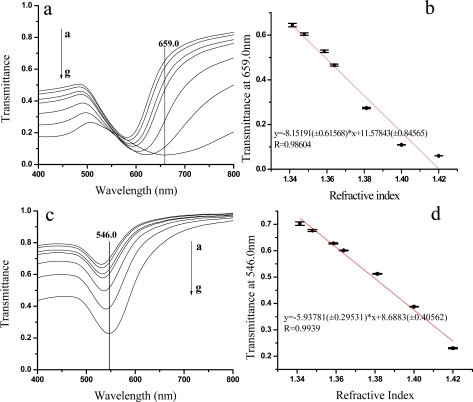
(a) and (c) The transmission spectra of different concentrations of sucrose solution by using the 48-nm and 23-nm Au NP films, respectively. a-g are from low to high concentrations. (b) The transmission at 659.07 nm of 48-nm Au as a function of the refractive index of sucrose solutions. (d) The transmission at 546.0 nm of 23-nm Au as a function of the refractive index of sucrose solutions.

The response of these Au NP optical fiber LSPR sensors to the bulk phase was inspected. Figure 4a,c present a series of transmission spectra of the LSPR sensors incubated with different concentrations of sucrose solutions. It can be seen that the red shift of the LSPR band positions took place and the transmission peak intensities reduced gradually with the concentrations of sucrose solutions increasing. The transmittance at 659.0 and 546.3 nm was chosen to plot the LSPR response to the refractive index of sucrose solutions (Figure 4b and d). The sensitivity and the linear correlation coefficient (R) of the LSPR sensor are 9.46 TU/RIU(R = 0.9860) for 48-nm Au and 5.94 TU/RIU (R = 0.9939) for 23-nm Au. According to the formula of A = −logT (A is the absorbance and T is the transmittance), the sensitivity expressed by the absorbance and R are 13.09 AU/RIU (R = 0.9678) for 48-nm Au and 5.85 AU/RIU (R = 0.9666) for 23-nm Au. Compared with the literatures, the sensitivity was 4.21 AU/RIU for the optical fiber LSPR sensor with the silane coupling agent assembling Au NPs [4], and 0.46 AU/RIU for the LSPR sensing film made by the silane coupling agent assembling Au NPs on glass substrates [17], 0.21 AU/RIU for the sensing film prepared by the trilayer PE assembling Au NPs on glass substrates [12]. Obviously, the sensitivity of present optical fiber LSPR sensor prepared by trilayer PE assembling Au NPs is much higher. This may be caused by the larger Au NP adsorption capacity due to a higher LSPR intensity relative to the silane modified LSPR sensors. The benefit of repeated reflecting light in an optical fiber may be another reason.

**Figure 5.**
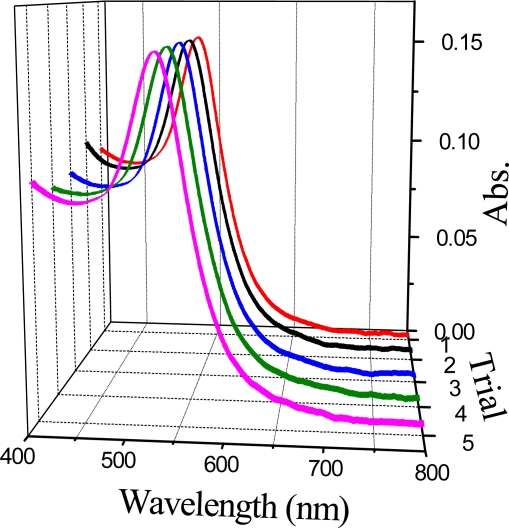
Comparison of the absorption spectra of the Au NP assembled films at different trials.

The reproducibility of the present optical fiber LSPR sensor was evaluated by the repetitious preparation of Au NP sensing films. Figure 5 shows the five absorption spectra of a 23-nm Au optical fiber LSPR sensor in water. Hardly any difference of these absorption curves can be seen. The standard deviation of LSPR peak intensity is only 0.02%. This illustrates that the sensor prepared by the assembly method is controllable and reproducible.

**Figure 6.**
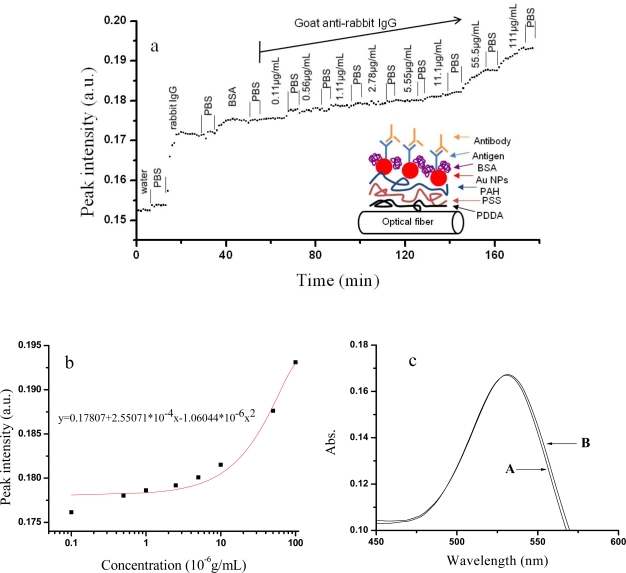
(a) The LSPR biosensing of different concentrations of goat anti-rabbit IgG (0.11–100 μg/mL). (b) The standard curve of LSPR peak intensities with the antigen concentration of goat anti-rabbit IgG. (c) The LSPR sensing by using the present setup before (A) and after (B) 11.1ng/mL of goat anti-rabbit IgG was added.

Here, the optical fiber LSPR sensor modified by rabbit IgG was employed to detect goat anti-rabbit IgG (insert in Figure 6a). Different concentrations of goat anti-rabbit IgG (in PBS solution) were pumped into a liquid cell. The 23-nm Au assembled optical fiber LSPR sensor was used here due to its shorter preparing time (Figure 2e). Figure 6a presents the dynamic process of goat anti-rabbit IgG adsorption. Both rabbit IgG and goat anti-rabbit IgG cause the peak intensities to remarkably increase. Bovine serum albumin (BSA) was used to block the unoccupied active spots. After pumping different concentrations of goat anti-rabbit IgG, the peak intensities elevated gradually with the goat anti-rabbit IgG concentration increasing. Figure 6b shows the relation between the peak intensities *versus* goat anti-rabbit IgG concentration. A working curve based on an exponential equation was obtained (insert in Figure 6b). Figure 6c is the LSPR spectra before (A) and after (B) a 200 μL of goat anti-rabbit IgG (100 ng/mL) were pumped into the liquid cell (the volume of 1.8 mL) and mixed adequately. The final concentration of goat anti-rabbit IgG equals to 11.1 ng/mL. The absorbance of curve B increased as 0.0003 unit. For the concentrations lower than 11.1 ng/mL, the present sensor had no visible response. So the lowest detection concentration of goat anti-rabbit IgG is supposed to be 11.1 ng/mL.

The anti-interference ability of the optical fiber LSPR sensor was inspected here. Goat anti-human IgG was pumped into the liquid cell instead of goat anti-rabbit IgG. The response of 100 μg/mL of mismatching goat anti-human IgG was similar to that of 0.1 μg/mL of matching goat anti-rabbit IgG (only differ from 0.0004 in intensity). Therefore, the fake positive for immunoassay by using the optical fiber LSPR sensor can be ignored except when the interfering concentration is 1,000-times of the analytes. This optical fiber sensor prepared by this method has the potential application as a portable immuno-sensor.

## 3. Experimental Section

### 3.1. Instruments and Reagents

Poly-diallyldimethylammonium chloride (PDDA, 20 wt% in water, *M*_w_ = 100,000−200,000) and poly-propyleneammonium chloride (PAH, *M*_w_ = 70 000) were purchased from Aldrich Co.. The polystyrene sulfonic acid sodium (PSS, *M*_w_ = 125 000) was obtained from Alfa Aesar Corporation. PDDA and PAH are the cationic PEs, and PSS is the anionic PE. Their structural formula is shown in Scheme 1. The PDDA solution was diluted to 0.5 wt% before use. The concentrations of PAH and PSS were both 1 mg/mL for PE assembly. The hydrochloro-auric acid (HAuCl_4_, gold content: ≥47.8%) was from Shanghai Chemical Reagent Co., Ltd. The citrate, sucrose, potassium dihydrogen phosphate (KH_2_PO_4_), sodium dihydrogen phosphate (NaH_2_PO_4_), sodium hydrogen phosphate (Na_2_HPO_4_), sodium chloride (NaCl), 40% hydrofluoric acid (HF) and the liquid paraffin were purchased from Beijing Chemical Factory and were all analytic-grade reagents without further purification. BSA, rabbit immunoglobulin G, goat anti-rabbit immunoglobulin G and goat anti-human immunoglobulin G were obtained from Beijing Dingguo Biological Technology Co., Ltd. Three-times deionized water was used in all experiments. The pH value of 0.01 mol/L phosphate buffer solution (PBS) is 7.33.

The Lamdba800 ultraviolet-visible spectrometer (Perkin Elmer Co.) was used to record the LSPR spectra of Au colloid. The JSM-6700F awkward silent launch scanning electronic microscope (FE-SEM, GEOL Co. Japan) was used to observe the appearance of Au NP assembled film.

### 3.2. Setup of Optical Fiber LSPR Sensor

The schematic drawing of the sensor is shown in Scheme 1. The sensing setup is composed of a bromine-tungsten lamp as a light source (the model: LS-1, Ocean Optic Co.), a light filter, an optical fiber coupler (Newport, Ocean Optic Co.), two optical fibers (NA = 0.22, P600-4 UV-VIS, length = ∼1.9 m, Ocean Optic Co.), a teflon liquid cell (length = 2 cm, volume = 1.8 mL), two 10× lenses, a XZ two-dimensional tunable optical fiber bracket, and a optical fiber spectrometer (the model: PC2000, Ocean Optic Co.). The fiber we used is composed of a pure silica core (diameter = 600 ± 10 μm) and a fluorine-doped silica cladding (thickness = 30 ± 3 μm).

A bromine-tungsten lamp provides a light in a region of 300∼1,000 nm. A light filter as an attenuator weakens the intensity of light source in a certain wavelength in present study, which can make the luminous intensity distribution more even. An optical fiber coupler couples the light into the incident optical fiber. Another optical fiber is connected to an optical fiber bracket and an optical fiber spectrometer. The emergent light passes through a 10× lens to focus on the end of optical fiber fixed in a two-dimensional tunable optical the fiber bracket, and then is transformed to the signals through a portable spectrometer. First we adjusted the distance between the optical fiber bracket and the 10× lens to insure the end of optical fiber aiming at the focal point of 10× lens. Secondly we adjusted the two-dimensional tunable optical fiber bracket to make the spectral intensity at 559.0 nm get to about 3,550. Thus, the coupling of light path is completed.

The operation software we used in the experiment is Oiibase32. The integration time for each spectrum is 3 ms and the integral scan is 100-times. The boxcar is set to 20. A dark spectrum and a reference spectrum were both recorded through a clean etched optical fiber immersed in water.

### 3.3. The Etching Process of Optical Fiber

Silica fiber has a unique feature that the refractive index of its core is higher than that of its cladding layer. The light transmission is confined in the core. For preparing an evanescent field emission LSPR sensor, the cladding layer must be removed and expose the core of an optical fiber. A 2 mL of 40% HF solution was dropped into the liquid cell for the etching treatment of the cladding layer. A liquid paraffin as a protector layer was added to prevent the SiF_4_ diffusion. The real-time spectra of etching process of an optical fiber were monitored to optimize the etching time.

### 3.4. The Sensing Film Preparation

The Au colloids were synthesized *via* the Frens method [18]. One mL (or 2 mL) of 1% sodium citrate solution was dropwise added into a 100 mL of 0.01 % HAuCl_4_ solution. The size of Au NPs was tuned by adjusting the amount of sodium citrate.

The method of PE self-assembly was utilized to prepare metal NP sensing films [12]. The assembly process was divided into three steps. First, the hydroxylation of an optical fiber was accomplished by immersing the optical fiber for 40 min in an hydroxylating agent, a mixed solution of concentrated sulfuric acid and hydrogen peroxide (v/v = 7:3). The optical fiber was then exhaustively rinsed by water. Secondly, PE assembly was performed by the layer-by-layer deposition. Three kinds of PE solution, 0.5% of PDDA, 1 mg/mL of PSS and 1 mg/mL of PAH, were alternately pumped into the liquid cell. The assembly time was 15 min for each assembly process. The optical fiber was rinsed by a lot of water before next step. Thirdly, Au colloids with different sizes were pumped into the liquid cell. The LSPR spectra of the assembly process were monitored. Once a certain value of the LSPR band intensity was achieved, the assembly process was stopped. We repeated the Au NP assembly process on flat quartz substrates for SEM characterization.

### 3.4. Detection of Bulk Phase Refractive Index

Different concentrations of sucrose solutions were pumped into the liquid cell after modifying the sensing film. The order was from the lowest to highest concentration. Each concentration was performed three times.

### 3.5. Immuno-modification of the Au NP Sensing Film

The PBS buffer solution was pumped into liquid cell before 100 μg/mL of rabbit immunoglobulin G (IgG) was employed. The immuno-modification process was real-time monitored. After 10 min, the adsorption was stable. The LSPR sensor was cleaned by a PBS buffer after each step. Then a 100 μg/mL of BSA solution was injected to block the unoccupied active sites. Different concentrations of goat anti-rabbit immunoglobulin G (goat anti-rabbit IgG) were determined.

## 4. Conclusions

This article utilized trilayer poly-electrolyte to assemble Au NPs sensing film in order to obtain a high quality LSPR optical fiber sensor. The optical fiber LSPR sensor has been employed to detect the changes of the bulk phase refractive index and the assays of antibodies. The sensitivities are 13.09 AU/RIU(R = 0.9678) for 48-nm Au and 5.85 AU/RIU (R = 0.9666) for 23-nm Au. The sensor has the virtues of good reproduction, rapid preparation and high sensitivity. It provides an approach for biological sample analysis and investigating interactions between biological molecules.
